# Bis[μ-(*E*)-*N*-(pyridin-3-yl­methyl­idene)hy­droxy­amine]-κ^2^ *N* ^1^:*N* ^3^;κ^2^ *N* ^3^:*N* ^1^-bis­{[(*E*)-*N*-(pyridin-3-yl­methyl­idene-κ*N*)hy­droxy­amine]­silver(I)} dinitrate

**DOI:** 10.1107/S1600536812045898

**Published:** 2012-11-28

**Authors:** Shan Gao, Seik Weng Ng

**Affiliations:** a Key Laboratory of Functional Inorganic Material Chemistry, Ministry of Education, Heilongjiang University, Harbin 150080, People’s Republic of China; b Department of Chemistry, University of Malaya, 50603 Kuala Lumpur, Malaysia; c Chemistry Department, Faculty of Science, King Abdulaziz University, PO Box 80203 Jeddah, Saudi Arabia

## Abstract

In the centrosymmetric dinuclear title Ag^I^ compound, [Ag_2_(C_6_H_6_N_2_O)_4_](NO_3_)_2_, the aromatic amine-coordinated Ag^I^ atom is further bridged by two hydroxyl­amine mol­ecules that use aromatic and oxime N atoms for bridging, and it exists in a distorted trigonal-planar geometry. In the crystal, the nitrate anions link to the dinuclear compound mol­ecules *via* O—H⋯O hydrogen bonds, generating a chain running along the *a*-axis direction.

## Related literature

For bis­(nicotinyl­aldehyde oxime)silver perchlorate, see: Xu *et al.* (2012 ▶) and for (nitrato)(picolinaldehyde oxime)silver, see: Abu-Youssef *et al.* (2010 ▶).
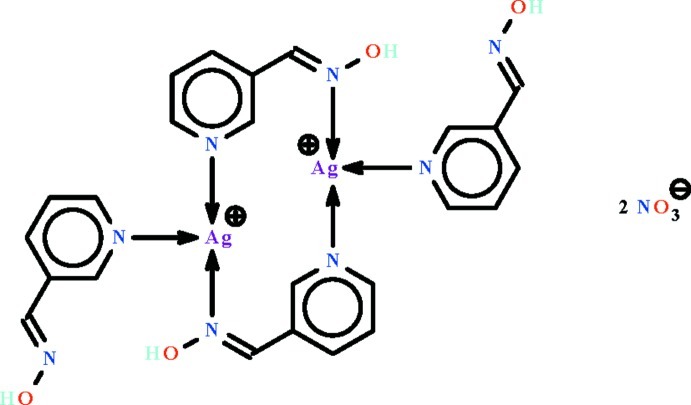


## Experimental

### 

#### Crystal data

[Ag_2_(C_6_H_6_N_2_O)_4_](NO_3_)_2_
*M* *_r_* = 828.27
Triclinic, 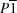

*a* = 7.2913 (10) Å
*b* = 8.3395 (10) Å
*c* = 13.1415 (17) Å
α = 92.934 (4)°
β = 95.008 (4)°
γ = 111.360 (3)°
*V* = 738.38 (16) Å^3^
*Z* = 1
Mo *K*α radiation
μ = 1.40 mm^−1^
*T* = 293 K
0.24 × 0.21 × 0.17 mm

#### Data collection

Rigaku R-AXIS RAPID IP diffractometer
Absorption correction: multi-scan (*ABSCOR*; Higashi, 1995 ▶) *T*_min_ = 0.730, *T*_max_ = 0.797
7293 measured reflections
3341 independent reflections
2896 reflections with *I* > 2σ(*I*)
*R*_int_ = 0.035

#### Refinement

*R*[*F* ^2^ > 2σ(*F* ^2^)] = 0.040
*wR*(*F* ^2^) = 0.107
*S* = 1.11
3341 reflections
210 parameters
H-atom parameters constrained
Δρ_max_ = 1.71 e Å^−3^
Δρ_min_ = −0.34 e Å^−3^

### 

Data collection: *RAPID-AUTO* (Rigaku, 1998 ▶); cell refinement: *RAPID-AUTO*; data reduction: *CrystalClear* (Rigaku/MSC, 2002 ▶); program(s) used to solve structure: *SHELXS97* (Sheldrick, 2008 ▶); program(s) used to refine structure: *SHELXL97* (Sheldrick, 2008 ▶); molecular graphics: *X-SEED* (Barbour, 2001 ▶); software used to prepare material for publication: *publCIF* (Westrip, 2010 ▶).

## Supplementary Material

Crystal structure: contains datablock(s) global, I. DOI: 10.1107/S1600536812045898/xu5646sup1.cif

Structure factors: contains datablock(s) I. DOI: 10.1107/S1600536812045898/xu5646Isup2.hkl

Additional supplementary materials:  crystallographic information; 3D view; checkCIF report

## Figures and Tables

**Table 1:** Hydrogen-bond geometry (Å, °)

*D*—H⋯*A*	*D*—H	H⋯*A*	*D*⋯*A*	*D*—H⋯*A*
O1—H1⋯O3^i^	0.84	2.39	3.189 (6)	158
O1—H1⋯O4^i^	0.84	2.20	2.925 (5)	145
O2—H2⋯O4^ii^	0.84	1.92	2.745 (4)	169

Symmetry codes: (i) 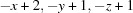; (ii) 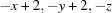.

**Fig. 1.**
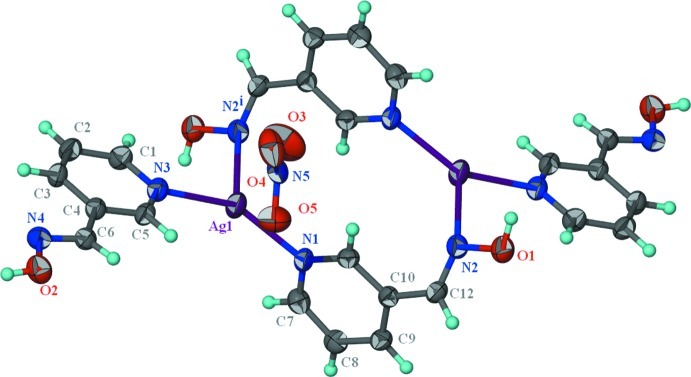
Thermal ellipsoid plot (Barbour, 2001) of [Ag(C6H6N2O)2]2 2NO3 at the 50% probability level; hydrogen atoms are drawn as spheres of arbitrary radius.

## supplementary crystallographic information

### Comment

A previous study on the adducts of silver salts with nicotinylaldehyde oxime reported the salt, [Ag(C_6_H_6_N_2_O)_2_] ClO_4_; the metal atom shows linear coordination (Xu *et al.*, 2012). The aromatic amine-coordinated Ag^I^ atom in dinuclear [Ag(C_6_H_6_N_2_O)_2_]_2_ 2NO_3_ is bridged by another hydroxylamine molecule that uses its aromatic and oxime N atoms for bridging, and it exists in a trigonal planar geometry (Scheme I, Fig. 1). The hydroxyl OH groups forms H atoms to the nitrate O atoms of adjacent molecules to generate a chain running along the shortest axis of the orthorhombic unit cell (Table 1).

With picolinylaldehyde oxime in place of nicotinylaldehyde oxime, the synthesis yields the monomeric adduct in which the metal atom is chelated by the ligand (Abu-Youssef *et al.*, 2010).

### Experimental

Nicotinyladehyde oxime was synthesized from the reaction of nicotinylaldehyde and hydroxylamine. Silver nitrate (1 mmol) dissolved in water (5 ml) was added to nicotinylaldehyde oxime (1 mmol) dissolved in ethanol (5 ml). The solution was filtered and set aside, away from light, for the growth of colorless crystals.

### Refinement

Carbon- and oxygen-bound H-atoms were placed in calculated positions (C–H 0.93 Å, O–H 0.84 Å) and were included in the refinement in the riding model approximation, with *U*(H) set to 1.2–1.5*U*(C,*O*).

The (-1 3 3), (5 - 10 1), (6 - 9 5) and (0 9 1) reflections were omitted.

The final difference Fourier map had a peak at 0.88 Å from Ag1.

### Crystal data

**Table:**

[Ag_2_(C_6_H_6_N_2_O)_4_](NO_3_)_2_	*Z* = 1
*M**_r_* = 828.27	*F*(000) = 412
Triclinic, *P*1	*D*_x_ = 1.863 Mg m^−^^3^
Hall symbol: -P 1	Mo *K*α radiation, λ = 0.71073 Å
*a* = 7.2913 (10) Å	Cell parameters from 6130 reflections
*b* = 8.3395 (10) Å	θ = 3.0–27.4°
*c* = 13.1415 (17) Å	µ = 1.40 mm^−^^1^
α = 92.934 (4)°	*T* = 293 K
β = 95.008 (4)°	Prism, colorless
γ = 111.360 (3)°	0.24 × 0.21 × 0.17 mm
*V* = 738.38 (16) Å^3^	

### Data collection

**Table:**

Rigaku R-AXIS RAPID IP diffractometer	3341 independent reflections
Radiation source: fine-focus sealed tube	2896 reflections with *I* > 2σ(*I*)
Graphite monochromator	*R*_int_ = 0.035
ω scan	θ_max_ = 27.4°, θ_min_ = 3.0°
Absorption correction: multi-scan (*ABSCOR*; Higashi, 1995)	*h* = −9→9
*T*_min_ = 0.730, *T*_max_ = 0.797	*k* = −10→10
7293 measured reflections	*l* = −17→16

### Refinement

**Table:**

Refinement on *F*^2^	Primary atom site location: structure-invariant direct methods
Least-squares matrix: full	Secondary atom site location: difference Fourier map
*R*[*F*^2^ > 2σ(*F*^2^)] = 0.040	Hydrogen site location: inferred from neighbouring sites
*wR*(*F*^2^) = 0.107	H-atom parameters constrained
*S* = 1.11	*w* = 1/[σ^2^(*F*_o_^2^) + (0.056*P*)^2^ + 0.3361*P*] where *P* = (*F*_o_^2^ + 2*F*_c_^2^)/3
3341 reflections	(Δ/σ)_max_ = 0.001
210 parameters	Δρ_max_ = 1.71 e Å^−^^3^
0 restraints	Δρ_min_ = −0.34 e Å^−^^3^

### Fractional atomic coordinates and isotropic or equivalent isotropic displacement parameters (Å^2^)

**Table:**

	*x*	*y*	*z*	*U*_iso_*/*U*_eq_	
Ag1	0.38614 (4)	0.61092 (3)	0.314850 (18)	0.04700 (13)	
O1	1.0366 (4)	0.7195 (3)	0.7651 (2)	0.0501 (6)	
H1	1.0758	0.6378	0.7541	0.075*	
O2	0.9485 (4)	1.1845 (3)	−0.0856 (2)	0.0522 (6)	
H2	0.9451	1.2272	−0.1419	0.078*	
O3	0.7359 (7)	0.5156 (5)	0.3151 (4)	0.1102 (16)	
O4	1.0106 (5)	0.6444 (5)	0.2602 (3)	0.0751 (10)	
O5	0.8581 (6)	0.7867 (4)	0.3354 (3)	0.0792 (10)	
N1	0.4261 (4)	0.7161 (4)	0.4767 (2)	0.0393 (6)	
N2	0.8633 (4)	0.6911 (4)	0.7003 (2)	0.0426 (6)	
N3	0.4227 (4)	0.6552 (4)	0.1507 (2)	0.0393 (6)	
N4	0.7795 (4)	1.0353 (4)	−0.0856 (2)	0.0411 (6)	
N5	0.8690 (4)	0.6504 (4)	0.3059 (2)	0.0436 (7)	
C1	0.2918 (5)	0.5528 (4)	0.0748 (3)	0.0420 (7)	
H1A	0.1875	0.4574	0.0915	0.050*	
C2	0.3047 (5)	0.5827 (4)	−0.0277 (3)	0.0438 (7)	
H2A	0.2104	0.5087	−0.0783	0.053*	
C3	0.4576 (5)	0.7221 (4)	−0.0537 (3)	0.0410 (7)	
H3	0.4689	0.7437	−0.1220	0.049*	
C4	0.5962 (5)	0.8315 (4)	0.0240 (2)	0.0354 (6)	
C5	0.5728 (5)	0.7911 (4)	0.1242 (2)	0.0366 (6)	
H5	0.6663	0.8621	0.1762	0.044*	
C6	0.7622 (5)	0.9865 (4)	0.0046 (3)	0.0375 (7)	
H6	0.8559	1.0499	0.0586	0.045*	
C7	0.3097 (5)	0.7957 (4)	0.5116 (3)	0.0430 (7)	
H7	0.1944	0.7853	0.4708	0.052*	
C8	0.3541 (5)	0.8913 (4)	0.6046 (3)	0.0429 (7)	
H8	0.2721	0.9467	0.6256	0.051*	
C9	0.5228 (5)	0.9041 (4)	0.6669 (3)	0.0407 (7)	
H9	0.5570	0.9704	0.7296	0.049*	
C10	0.6402 (5)	0.8177 (4)	0.6350 (2)	0.0361 (6)	
C11	0.5871 (5)	0.7270 (4)	0.5384 (2)	0.0369 (6)	
H11	0.6675	0.6713	0.5156	0.044*	
C12	0.8170 (5)	0.8228 (4)	0.6996 (2)	0.0396 (7)	
H12	0.8952	0.9234	0.7401	0.048*	

### Atomic displacement parameters (Å^2^)

**Table:**

	*U*^11^	*U*^22^	*U*^33^	*U*^12^	*U*^13^	*U*^23^
Ag1	0.05390 (19)	0.0543 (2)	0.03057 (17)	0.01848 (14)	0.00080 (12)	0.00222 (11)
O1	0.0459 (13)	0.0570 (15)	0.0475 (15)	0.0220 (12)	−0.0050 (11)	0.0026 (11)
O2	0.0459 (13)	0.0490 (14)	0.0565 (16)	0.0103 (11)	0.0045 (12)	0.0153 (12)
O3	0.101 (3)	0.062 (2)	0.155 (5)	0.007 (2)	0.035 (3)	0.043 (3)
O4	0.0682 (19)	0.124 (3)	0.062 (2)	0.064 (2)	0.0219 (16)	0.034 (2)
O5	0.100 (3)	0.0675 (19)	0.079 (2)	0.0441 (19)	0.009 (2)	−0.0041 (16)
N1	0.0458 (14)	0.0412 (14)	0.0277 (13)	0.0131 (12)	0.0010 (11)	0.0040 (10)
N2	0.0430 (14)	0.0408 (14)	0.0401 (15)	0.0129 (12)	−0.0037 (12)	0.0043 (11)
N3	0.0400 (14)	0.0462 (15)	0.0333 (14)	0.0179 (12)	0.0039 (11)	0.0034 (11)
N4	0.0401 (14)	0.0401 (14)	0.0439 (16)	0.0154 (12)	0.0063 (12)	0.0039 (12)
N5	0.0483 (16)	0.0521 (17)	0.0383 (15)	0.0257 (15)	0.0081 (13)	0.0138 (13)
C1	0.0393 (16)	0.0407 (17)	0.0425 (18)	0.0106 (14)	0.0054 (14)	0.0021 (13)
C2	0.0442 (17)	0.0446 (17)	0.0386 (18)	0.0150 (15)	−0.0037 (14)	−0.0056 (14)
C3	0.0471 (18)	0.0475 (18)	0.0297 (15)	0.0197 (15)	0.0012 (14)	0.0024 (13)
C4	0.0349 (14)	0.0415 (16)	0.0328 (15)	0.0178 (13)	0.0035 (12)	0.0027 (12)
C5	0.0352 (15)	0.0437 (16)	0.0310 (15)	0.0163 (13)	−0.0002 (12)	−0.0023 (12)
C6	0.0367 (15)	0.0424 (16)	0.0373 (17)	0.0203 (14)	0.0013 (13)	0.0012 (13)
C7	0.0394 (16)	0.0468 (17)	0.0443 (19)	0.0166 (15)	0.0061 (14)	0.0098 (14)
C8	0.0470 (17)	0.0457 (18)	0.0413 (18)	0.0210 (15)	0.0138 (15)	0.0082 (14)
C9	0.0530 (19)	0.0390 (16)	0.0322 (16)	0.0188 (15)	0.0093 (14)	0.0022 (12)
C10	0.0424 (16)	0.0309 (14)	0.0319 (15)	0.0097 (13)	0.0039 (13)	0.0057 (11)
C11	0.0439 (16)	0.0315 (14)	0.0356 (16)	0.0141 (13)	0.0066 (13)	0.0021 (12)
C12	0.0455 (17)	0.0385 (16)	0.0315 (16)	0.0129 (14)	0.0015 (13)	−0.0022 (12)

### Geometric parameters (Å, º)

**Table:**

Ag1—N1	2.212 (3)	C2—C3	1.369 (5)
Ag1—N3	2.229 (3)	C2—H2A	0.9300
Ag1—N2^i^	2.498 (3)	C3—C4	1.396 (5)
O1—N2	1.397 (4)	C3—H3	0.9300
O1—H1	0.8400	C4—C5	1.385 (4)
O2—N4	1.396 (4)	C4—C6	1.466 (5)
O2—H2	0.8400	C5—H5	0.9300
O3—N5	1.208 (5)	C6—H6	0.9300
O4—N5	1.255 (4)	C7—C8	1.370 (5)
O5—N5	1.214 (4)	C7—H7	0.9300
N1—C11	1.339 (4)	C8—C9	1.383 (5)
N1—C7	1.349 (5)	C8—H8	0.9300
N2—C12	1.262 (4)	C9—C10	1.381 (5)
N2—Ag1^i^	2.498 (3)	C9—H9	0.9300
N3—C1	1.337 (5)	C10—C11	1.392 (4)
N3—C5	1.344 (4)	C10—C12	1.466 (5)
N4—C6	1.274 (4)	C11—H11	0.9300
C1—C2	1.387 (5)	C12—H12	0.9300
C1—H1A	0.9300		
			
N1—Ag1—N3	149.49 (11)	C5—C4—C3	117.7 (3)
N1—Ag1—N2^i^	107.79 (10)	C5—C4—C6	119.0 (3)
N3—Ag1—N2^i^	101.48 (10)	C3—C4—C6	123.3 (3)
N2—O1—H1	109.5	N3—C5—C4	123.8 (3)
N4—O2—H2	109.5	N3—C5—H5	118.1
C11—N1—C7	117.6 (3)	C4—C5—H5	118.1
C11—N1—Ag1	119.9 (2)	N4—C6—C4	120.7 (3)
C7—N1—Ag1	121.6 (2)	N4—C6—H6	119.6
C12—N2—O1	112.4 (3)	C4—C6—H6	119.6
C12—N2—Ag1^i^	123.3 (2)	N1—C7—C8	122.8 (3)
O1—N2—Ag1^i^	115.10 (18)	N1—C7—H7	118.6
C1—N3—C5	117.2 (3)	C8—C7—H7	118.6
C1—N3—Ag1	121.6 (2)	C7—C8—C9	119.0 (3)
C5—N3—Ag1	121.1 (2)	C7—C8—H8	120.5
C6—N4—O2	110.6 (3)	C9—C8—H8	120.5
O3—N5—O5	120.1 (4)	C10—C9—C8	119.5 (3)
O3—N5—O4	117.9 (4)	C10—C9—H9	120.3
O5—N5—O4	121.9 (4)	C8—C9—H9	120.3
N3—C1—C2	122.8 (3)	C9—C10—C11	117.8 (3)
N3—C1—H1A	118.6	C9—C10—C12	121.5 (3)
C2—C1—H1A	118.6	C11—C10—C12	120.6 (3)
C3—C2—C1	119.4 (3)	N1—C11—C10	123.3 (3)
C3—C2—H2A	120.3	N1—C11—H11	118.4
C1—C2—H2A	120.3	C10—C11—H11	118.4
C2—C3—C4	119.0 (3)	N2—C12—C10	120.1 (3)
C2—C3—H3	120.5	N2—C12—H12	119.9
C4—C3—H3	120.5	C10—C12—H12	119.9
			
N3—Ag1—N1—C11	−93.9 (3)	O2—N4—C6—C4	−179.6 (3)
N2^i^—Ag1—N1—C11	103.1 (2)	C5—C4—C6—N4	−174.4 (3)
N3—Ag1—N1—C7	75.2 (3)	C3—C4—C6—N4	4.9 (5)
N2^i^—Ag1—N1—C7	−87.8 (3)	C11—N1—C7—C8	2.6 (5)
N1—Ag1—N3—C1	−147.5 (3)	Ag1—N1—C7—C8	−166.8 (2)
N2^i^—Ag1—N3—C1	16.0 (3)	N1—C7—C8—C9	−1.5 (5)
N1—Ag1—N3—C5	29.4 (4)	C7—C8—C9—C10	−1.4 (5)
N2^i^—Ag1—N3—C5	−167.1 (2)	C8—C9—C10—C11	2.9 (4)
C5—N3—C1—C2	−0.6 (5)	C8—C9—C10—C12	−178.1 (3)
Ag1—N3—C1—C2	176.4 (3)	C7—N1—C11—C10	−0.9 (5)
N3—C1—C2—C3	0.2 (5)	Ag1—N1—C11—C10	168.7 (2)
C1—C2—C3—C4	−0.4 (5)	C9—C10—C11—N1	−1.8 (5)
C2—C3—C4—C5	0.9 (5)	C12—C10—C11—N1	179.2 (3)
C2—C3—C4—C6	−178.4 (3)	O1—N2—C12—C10	179.4 (3)
C1—N3—C5—C4	1.2 (5)	Ag1^i^—N2—C12—C10	−35.6 (4)
Ag1—N3—C5—C4	−175.8 (2)	C9—C10—C12—N2	144.0 (3)
C3—C4—C5—N3	−1.4 (5)	C11—C10—C12—N2	−37.1 (5)
C6—C4—C5—N3	178.0 (3)		

Symmetry code: (i) −*x*+1, −*y*+1, −*z*+1.

### Hydrogen-bond geometry (Å, º)

**Table:**

*D*—H···*A*	*D*—H	H···*A*	*D*···*A*	*D*—H···*A*
O1—H1···O3^ii^	0.84	2.39	3.189 (6)	158
O1—H1···O4^ii^	0.84	2.20	2.925 (5)	145
O2—H2···O4^iii^	0.84	1.92	2.745 (4)	169

Symmetry codes: (ii) −*x*+2, −*y*+1, −*z*+1; (iii) −*x*+2, −*y*+2, −*z*.

## References

[bb1] Abu-Youssef, M. A., Soliman, S. V., Langer, V., Gohar, Y. M., Hasanen, A. A., Makhyoun, M. A., Zaky, A. H. & Öhrström, L. R. (2010). *Inorg. Chem.* **49**, 9788–9797. 10.1021/ic100581k 20929250

[bb2] Barbour, L. J. (2001). *J. Supramol. Chem.* **1**, 189–191.

[bb3] Higashi, T. (1995). *ABSCOR* Rigaku Corporation, Tokyo, Japan.

[bb4] Rigaku (1998). *RAPID-AUTO* Rigaku Corporation, Tokyo, Japan.

[bb5] Rigaku/MSC (2002). *CrystalClear* Rigaku/MSC Inc., The Woodlands, Texas, USA.

[bb6] Sheldrick, G. M. (2008). *Acta Cryst.* A**64**, 112–122. 10.1107/S0108767307043930 18156677

[bb7] Westrip, S. P. (2010). *J. Appl. Cryst.* **43**, 920–925.

[bb8] Xu, J., Gao, S., Ng, S. W. & Tiekink, E. R. T. (2012). *Acta Cryst.* E**68**, m735–m736. 10.1107/S1600536812019290 PMC3379077 22719298

